# Strengthening antimicrobial resistance surveillance across African military settings

**DOI:** 10.1093/inthealth/ihaf102

**Published:** 2025-09-12

**Authors:** Yusuff Adebayo Adebisi

**Affiliations:** College of Social Sciences, University of Glasgow, 40 Bute Gardens, Glasgow G12 8RT, UK; Global Health Focus, Abuja, Nigeria

**Keywords:** Africa, antimicrobial resistance, military healthcare, surveillance

## Abstract

While antimicrobial resistance (AMR) surveillance within civilian health systems has progressed, military health systems remain under-represented in related research and policy frameworks. Military personnel, particularly during deployments, often operate in environments characterised by combat-related injuries, overcrowding and inadequate sanitation, which increase the risk of spread and emergence of drug-resistant infections. Despite these vulnerabilities, national AMR strategies in Africa largely prioritise civilian systems, with minimal integration of military health surveillance data or clinical insights from military medical facilities. As a result, AMR trends within military populations and conflict zones remain poorly documented. This paper examines the distinctive AMR challenges in African military settings and proposes strategies for incorporating military medical surveillance data into national AMR frameworks. Drawing on international models, such as the US Armed Forces Health Surveillance Division's Antimicrobial Resistance Monitoring and Research programme, it highlights the potential for military facilities to serve as sentinel sites for resistance monitoring. Integrating military medical surveillance data into national systems would enhance understanding of resistance patterns and support more inclusive and effective containment strategies, while strengthening antimicrobial stewardship within military health facilities to reduce inappropriate antimicrobial use.

Antimicrobial resistance (AMR) is a major public health concern in Africa, exacerbated by the combined burden of infectious diseases, non-communicable conditions and injuries.^1–3^ While surveillance in civilian healthcare has expanded through initiatives such as the Mapping Antimicrobial Resistance and Antimicrobial Use Partnership (MAAP),^4^ military settings remain largely unexamined. For the purposes of this paper, ‘military settings’ refers to the full range of environments in which armed forces personnel live, work and receive healthcare, including military health facilities (field hospitals, base clinics), deployment environments (conflict zones, peacekeeping missions, training camps), living quarters (barracks, temporary camps) and operational theatres where combat or humanitarian assistance takes place. Military personnel face distinct drug-resistant infection risks due to combat-related trauma, overcrowded barracks and deployments in areas with poor sanitation.^5,6^ These conditions are intensified by operational demands and may facilitate the emergence and spread of resistant pathogens.

Despite these risks, national AMR strategies and the African Union Framework for AMR Control (2020–2025) focus almost entirely on civilian systems.^7^ To the best of my knowledge, there has been no prior Africa-wide synthesis of AMR in military settings, leaving a critical gap in the evidence base. Neglecting AMR data from military settings creates blind spots in national and regional surveillance, allowing resistant pathogens circulating among armed forces to spread undetected into civilian populations and across borders, which may undermine broader containment efforts.

Military settings in Africa may contribute to AMR due to context-specific risk factors. As summarised in Table 1, these environments expose personnel to a range of priority pathogens, from *Acinetobacter baumannii* in combat wounds to *Vibrio cholerae* in humanitarian deployments. Combat injuries, which are common in conflict zones, often require prolonged antibiotic use, a recognised driver of resistance.^8–10^ Armed conflict increases the incidence of trauma requiring antibiotic treatment and disrupts medical supply chains, infection-prevention practices and laboratory services, thereby compounding the risk of resistant infections.^10^ Surveillance gaps make it difficult to quantify antimicrobial misuse or track resistance trends.

**Table 1.** Potential target pathogens for AMR surveillance in African military settings

Pathogen	Clinical significance	Military-relevant settings	Notes
*Acinetobacter baumannii*	Causes wound and bloodstream infections; frequently MDR	Field hospitals treating combat injuries	Common in military wounds
Methicillin-resistant *Staphylococcus aureus* (MRSA)	Skin, soft tissue and invasive infections; colonisation risk	Barracks, training camps	High transmission in crowded living quarters
*Escherichia coli* (ESBL-producing)	Urinary and bloodstream infections; ESBL producers	Deployed personnel in sanitation-poor field conditions	Reflects local enteric resistance patterns
*Klebsiella pneumoniae* (CRE)	Pneumonia, wound and bloodstream infections; carbapenem-resistant	ICUs in mobile hospitals	Emerging CRE clones threaten treatment options
*Pseudomonas aeruginosa*	Wound and respiratory infections; often MDR	Burn units, field ICUs	Environmental organism, thrives in moist settings
*Mycobacterium tuberculosis*	Pulmonary TB; MDR-TB increasing	Barracks, peacekeeping deployments	High TB burden in many deployment regions
*Salmonella enterica* (typhoidal)	Typhoid fever; potential outbreaks in camps	Field kitchens, large troop gatherings	Indicator of hygiene and water quality
*Vibrio cholerae*	Cholera outbreaks; rapid spread in displaced populations	Refugee camps, areas with disrupted water/sanitation infrastructure	Sentinel of severe waterborne risk
*Staphylococcus pseudintermedius*/*Escherichia coli* (veterinary)	Opportunistic and zoonotic infections in humans; can be MDR	Military working dogs, horses and livestock in operational bases	Highlights One Health AMR risks from animal–human interfaces

AMR: antimicrobial resistance; CRE: Carbapenem-resistant Enterobacteriaceae; ESBL: extended-spectrum β-lactamase; MDR: multidrug-resistant.

Where military-specific data are available, they highlight important concerns. For example, a recent study in Nigerian Navy hospitals found antibiotics prescribed in 46.3% of encounters, exceeding the WHO/International Network for Rational Use of Drugs (WHO/INRUD) threshold of <30%.^11^ Similarly, an analysis of 11 590 prescriptions across three Nigerian military hospitals reported antibiotic use well above WHO/INRUD reference limits, with significantly higher rates during the COVID-19 period compared with prepandemic levels.^12^ Further evidence from Military Hospital Lagos showed that 93.8% of respiratory tract infection cases were upper respiratory, yet only 3% of patients underwent laboratory testing before antibiotic prescription, underlining diagnostic gaps that can drive unnecessary antibiotic use.^13^ These findings highlight substantial antibiotic prescribing pressures in African military facilities, which may be even greater in conflict-affected or resource-limited settings.^10^ Without targeted stewardship and surveillance interventions, these prescribing patterns risk accelerating the emergence and spread of multidrug-resistant pathogens within and beyond military populations.

Furthermore, overcrowded barracks can increase transmission risk, while troop movements across borders may facilitate regional spread. In addition, military operations may also involve the care and management of animals, including working dogs, horses and, in some contexts, livestock, which can introduce additional AMR risks through veterinary antibiotic use and potential zoonotic transmission. These animal–human interfaces in military settings reflect One Health concerns and underline the need for integrated surveillance approaches. Civil–military transmission can also occur when military hospitals treat civilian patients during emergencies,^11^ or when demobilised personnel return to densely populated urban areas, potentially carrying resistant organisms acquired during deployment. International data show that multidrug-resistant organisms (MDROs), including *A. baumannii*, are prevalent in combat-related wounds from Iraq and Afghanistan,^14^ raising concerns about similar patterns in African deployments. Military personnel often operate in areas with high burdens of TB, malaria and cholera, potentially compounding exposure to resistant pathogens. In the absence of targeted surveillance, the contribution of African military settings to AMR transmission remains poorly defined. Closing this gap is necessary to inform both military and public health strategies.

Africa's AMR national action plans, aligned with WHO and Africa Centres for Disease Control and Prevention (CDC) frameworks, primarily address civilian healthcare systems, with limited consideration for military contexts.^15,16^ Where military health policies do exist, they are often not publicly accessible, and data on service members or veterans are seldom integrated into national surveillance efforts.^17^ This omission may reflect both limited resources and the prioritisation of civilian systems due to their broader population coverage. However, military personnel interact regularly with civilian populations during and after deployments, creating potential pathways for transmission of resistant pathogens. Integrating these data streams will require addressing security concerns, protecting sensitive information and ensuring interoperability between civilian and military surveillance platforms. Including military settings in AMR surveillance could improve data completeness and support more comprehensive national response strategies.

A 2017 review identified 144 studies on AMR prevalence and susceptibility patterns in human bacterial pathogens across Africa, none of which focused specifically on military contexts.^18^ Research in this area is constrained by the restricted availability of military health data, which are often classified. Surveillance may also be hindered by military operations, which can disrupt laboratory activities and data collection, compounding the evidence gap. Even within civilian systems, diagnostic capacity is severely limited; the MAAP's 14-country analysis found that only 1.3% of 50 000 laboratories could perform bacteriology,^4^ suggesting that military medical facilities may face even greater limitations. In the absence of data from military facilities, national estimates rely on civilian proxies that may not accurately represent military operational environments. Closing this evidence gap is essential to determine whether military settings contribute meaningfully to AMR transmission and to guide the development of targeted intervention strategies. Early steps could include piloting sentinel AMR surveillance sites in selected military bases, beginning with peacekeeping operations and high-turnover training camps, before scaling to national coverage.

International experience underlines the importance of AMR surveillance in military settings. In the USA, the Antimicrobial Resistance Monitoring and Research (ARMoR) programme tracks MDROs across military facilities and has documented resistance trends in deployed personnel.^19^ European studies report Enterobacteriaceae colonisation in 4.7% of returning German soldiers, linking deployment to acquisition of resistant pathogens.^20^ These findings illustrate that military environments, characterised by trauma, high antibiotic use and frequent mobility, can facilitate AMR transmission. Military personnel often serve in regions with high infectious disease burdens and may reflect local resistance profiles.^21,22^ The use of broad-spectrum antibiotics is common in military healthcare and is often empirical due to limited diagnostic capacity.^23–26^ However, data on prescribing practices and resistance outcomes in African military contexts are lacking. The US Military Health System's experience with inconsistent diagnostic testing suggests that similar or greater challenges likely exist in under-resourced African settings.^27^ Targeted surveillance could help characterise AMR patterns, identify emerging clones such as carbapenem-resistant Enterobacterales and inform regionally adapted responses. Key policy actions to address these gaps are summarised in Table 2.

**Table 2.** Recommendations for strengthening AMR surveillance in African military settings

Recommendation	Rationale	Potential implementing partners
1. Establish a military AMR task force	Coordinates surveillance, standardises data and facilitates civil–military collaboration	Ministries of defence, ministries of health, Africa CDC
2. Integrate military health data into national AMR plans	Ensures that AMR data from soldiers and field hospitals contribute to national and regional strategies	National AMR coordinating committees, WHO, Africa CDC
3. Deploy mobile diagnostic laboratories in conflict zones and barracks	Enhances real-time detection of resistant pathogens where conventional laboratories are unavailable	Military medical corps, Africa CDC, international NGOs
4. Conduct targeted operational research on AMR in military settings	Fills key evidence gaps on pathogen prevalence, antimicrobial use and transmission risks	Military research units, academic institutions, public health agencies
5. Build laboratory capacity within military health facilities	Improves in situ bacteriological testing and reduces reliance on empirical antibiotic use	Ministries of defence, Global Fund, AMR Donor Consortium
6. Include military personnel in regional AMR training and stewardship programmes	Aligns prescribing behaviour with national guidelines and promotes shared best practices	Africa CDC, WHO, regional training institutions
7. Monitor AMR risks among peacekeeping and returning personnel	Addresses transmission pathways between military and civilian populations	AU Peace and Security Department, national AMR programmes
8. Secure dedicated funding for AMR surveillance in military settings	Ensures sustainable implementation of surveillance, capacity building and operational research	Ministries of defence, Africa CDC, African Development Bank, AMR Multi-Partner Trust Fund, bilateral donors

AMR: antimicrobial resistance; AU: African Union; CDC: Centres for Disease Control and Prevention; NGO: non-government organisation.

Global initiatives such as the US ARMoR programme offer a potential model for Africa. A dedicated military AMR task force could be established in collaboration with the Africa CDC to support integration of military health data into national surveillance systems. Without such coordination, gaps in AMR reporting will persist, limiting the effectiveness of regional containment strategies. Expanding surveillance to include military facilities, particularly those supporting peacekeeping operations, would improve data coverage and inform more comprehensive policy responses.

## Conclusion

Military settings in Africa remain a neglected component of AMR surveillance. Integrating data from these contexts into national systems could improve completeness and help identify resistance trends that may otherwise go undetected. Lessons from other regions suggest that military facilities can complement civilian surveillance networks, but implementation will require careful consideration of security, interoperability and ethical data governance. Incremental, well-coordinated approaches could strengthen both military and civilian AMR responses across the continent. To close this gap, the Africa CDC should formally incorporate military health surveillance into the next African Union AMR framework and require its integration into country-level National Action Plans, ensuring that military data collection, analysis and reporting are embedded in both national and regional strategies from the outset.

## Data Availability

Not applicable.
